# Neutrophil to lymphocyte ratio (NLR) and short-term mortality risk in elderly acute medical patients admitted to a University Hospital Emergency Department

**DOI:** 10.1007/s11739-024-03683-8

**Published:** 2024-06-25

**Authors:** Gioacchino Galardo, Luca Crisanti, Andrea Gentile, Marco Cornacchia, Francesca Iatomasi, Iacopo Egiddi, Emanuele Puscio, Danilo Menichelli, Francesco Pugliese, Daniele Pastori, and the Research on Medical patients Admitted to the Emergency Department (ROMA-ED) investigators

**Affiliations:** 1 Department of Clinical Internal, Anesthesiological and Cardiovascular Sciences, Sapienza University of Rome, Piazzale Aldo Moro 5, 00185 Rome, Italy; 2 Postgraduate School of Emergency Medicine, Sapienza University of Rome, 00189 Rome, Italy; 3 IRCCS Neuromed, Località Camerelle, 86077 Pozzilli, IS Italy; 4 Department of General Surgery and Surgical Specialties Paride Stefanini, Sapienza University of Rome, Rome, Italy; 5 Medical Emergency Unit, Policlinico Umberto I, Rome, Italy

**Keywords:** Neutrophil-to-lymphocyte ratio (NLR), Mortality, Emergency department (ED)

## Abstract

Early identification of patients with a poorer prognosis in the Emergency Department (ED) is crucial for prompt treatment and resource allocation. We investigated the relationship between the Neutrophil to Lymphocyte Ratio (NLR) and 30-day mortality in elderly acute medical patients. Prospective single-center cohort study including consecutive patients admitted to the ED. Inclusion criteria were age > 65 years and medical condition as the cause of ED access. Exclusion criteria were patients admitted for traumatic injuries or non-traumatic surgical diseases. ROC analysis was used to set the best cut-off of the NLR for mortality. 953 patients were included and 142 (14.9%) died during follow-up. ROC analysis showed a good predictive value of the NLR with an AUC 0.70, 95%CI 0.67–0.73 (*p* < 0.001) and identified a NLR > 8 as the best cut-off. Patients with NLR > 8 had a more serious triage code (72.6% had a triage code ≤ 2) and an increased heart rate and body temperature. They more often presented with dyspnea, abdominal pain, falls and vomiting. They also were characterized by an increase in urea, creatinine, white blood cells, neutrophils, fibrinogen, D-dimer, glycemia, CRP, LDH and transaminases and by a decrease in eGFR, of lymphocytes and monocytes. Multivariable logistic regression analysis demonstrated that the NLR remained associated with mortality after adjustment for confounders (Odds ratio 2.563, 95%CI 1.595–4.118, *p* < 0.001). Patients with NLR > 8 showed a higher mortality rate. NLR is an easy and inexpensive tool that may be used for risk stratification in the ED. The results of this study need to be validated in larger external cohorts.

## Introduction

In today’s healthcare landscape, one of the paramount challenges facing emergency departments (EDs) is the accurate risk stratification of elderly patients. As the population ages, EDs are witnessing a growing influx of older adults seeking medical attention, often presenting with complex and multifaceted health issues. Effectively assessing the risk associated with these patients is crucial, as it directly impacts clinical decision-making, resource allocation, and the overall quality of care provided [1–3]. In-hospital mortality for patients older than 65 was found to be between 8.2% and 22.8% when admitted for acute conditions [4–6].

Currently the risk stratification is performed through clinical scores that are specific for each disease (i.e., SOFA score, Ranson’s criteria), but general criteria that can help the clinician identify patients at high risk of death are lacking. As the rapid identification of high-risk patients is crucial for the early initiation of evidence-based life-saving treatment and management, new scores and biomarkers are being tested with this aim.

One of those is the neutrophil-to-lymphocyte ratio (NLR), which is a simple and inexpensive score calculated as a ratio between the neutrophil and lymphocyte counts. NLR is a biomarker that takes into account two faces of the immune system: the innate immune response, mainly due to neutrophils, and adaptive immunity, supported by lymphocytes [7, 8]. Several studies reported the prognostic value of NLR and its role as a marker of systemic inflammations in numerous pathologies such as chronic obstructive pulmonary disease (COPD), atrial fibrillation, coronary artery disease (CAD), sepsis, and acute pancreatitis [9–21]. Furthermore, the kinetic of this biomarker is really fast, NLR is thought to change within 6 h of the onset of physiologic stress, far earlier than other markers such as white cell count or C-reactive protein (CRP)[22].

Given those premises and the association with poor outcomes in different conditions, NLR could be useful in risk stratification of patients presenting to the ED with non-surgical conditions.

The aim of this study was to assess the value in the risk stratification of the NLR, its capability to predict all-cause mortality in patients presenting to the ED and establish a cut-off for elderly acute medical patients.

## Methods

### Study design and population

This was a single centre, prospective, observational study which included consecutive patients presenting to the ED of the Policlinico Universitario Umberto I in Rome between the 28th of April and the 28th of May 2022. Adult patients older than 65 years old presenting to the ED were enrolled after providing written informed consent. Patients presenting for a traumatic injury or non-traumatic surgical diseases and SARS-CoV-2 positive patients were excluded from the study. The study was carried out according to the principles of the Declaration of Helsinki and approved by the Sapienza University of Rome Ethics committee (Prot. 0405/2022). Reporting is in accordance with the STROBE statement. The authors designed the study, gathered, and analyzed the data anonymously, vouched for the data and analysis, wrote the manuscript, and decided to submit it for publication.

### Relative NLR calculation

The relative neutrophil–lymphocyte ratio (NLR) was calculated as follows: (% neutrophil count, cells/μL)/(% lymphocyte count, cells/μL).

### Routine clinical assessment

All patients underwent clinical assessment that included medical history, physical examination, standard blood tests, 12-lead electrocardiogram (ECG) and vital parameters at admission, everything was documented on the medical records. ED discharge diagnosis was prospectively documented. Management of patients was left to the discretion of the attending physician.

### Triage and priority codes

At entry into the ED, patients are assigned a code with decremental priority ranging from 1 to 5 depending on their severity. In detail: *Code 1:* interruption or impairment of one or more vital functions with immediate access; *Code 2*: risk of impairment of vital functions. Condition with developmental risk or severe pain access within 15 min; *Code 3*: deferrable urgency stable condition without developmental risk with suffering and effect on the general state which usually requires complex services access within 60 min; *Code 4*: green minor urgency stable condition without developmental risk which usually requires simple single specialist diagnostic therapeutic services access within 120 min; *Code 5*: non-urgent problem not urgent or of minimum clinical relevance access within 240 min (source Italian Ministry of Health website: https://www.salute.gov.it/imgs/C_17_pubblicazioni_3145_allegato.pdf).

### Follow-up and clinical endpoints

Information regarding death during follow-up were obtained from the patient’s hospital records and the national death registry. The primary endpoint was all-cause mortality at 30 days.

### Statistical analysis

Continuous variables are presented as average and standard deviation (SD) or median and interquartile range [IQR] and compared using the student *t*- test or the Mann–Whitney *U* test as appropriate. Categorical variables are presented as count and percentages (%) and compared with the Pearson χ^2^ test or Fisher exact test, as appropriate.

The descriptive analysis was performed by comparing patients with a NLR below or above 8 and according to the vital status at 30 days. To calculate the odds ratio (OR) and its 95% confidence intervals (95% CI) for all-cause mortality at 30 days a univariable and multivariable logistic regression analysis was performed. In the multivariable model, we inserted significant variables from univariable regression analysis.

In detail, we inserted the following variables into the multivariable model: relative NLR > 8, female sex, age, SBP, shock, respiratory rate, swollen legs, atrial fibrillation (AF), altered mental status, active cancer, blood glucose, GOT/AST, hemoglobin, creatinine, LDH, and mean platelet volume (MPV). Other significant variables were not inserted if collinear (as an example total WBC, neutrophil or lymphocytes, CRP with NLR, dyspnoea with respiratory rate, insulin use with blood glucose).

We also built the receiver operating characteristic (ROC) curve with the value of the Youden index to test the predictive value of the NLR in the whole population and in the subgroup of women.

All hypothesis testing was two-tailed and *P* values of less than 0.05 were considered to indicate statistical significance without adjustments for multiple testing. Statistical analyses were performed using IBM SPSS 25 and MedCalc.

## Results

### Study population and characteristics

We included 1074 consecutive patients. Of these, 121 were excluded due to lack of personal data or for not having performed blood tests before discharge. Therefore, 953 patients remained available for analysis. Out of those, 267 (28%) had an NLR > 8.

The characteristics of the population, stratified by NLR values above or below 8, are shown in Table 1.

**Table 1:** Characteristics of the study population according to the NLR values

	Whole cohort (*n* = 953)	Patients with NLR ≤ 8 (*n* = 686)	Patients with NLR > 8 (*n* = 267)	*P* value
*Triage code* (%)				0.002
1	201 (21.1)	133 (19.4)	67 (25.2)	
2	419 (43.9)	292 (42.5)	127 (47.4)	
3	292 (30.7)	223 (32.5)	70 (26.3)	
4	41 (4.3)	38 (5.6)	3 (1.1)	
Women (%)	474 (49.7)	350 (51)	124(46.4)	n.s
Age (years)	79.1 ± 8.1	78.6 ± 7.9	80.2 ± 8.4	0.004
SBP (mmHg)	133.8 ± 22.9	135.1 ± 22.7	130.5 ± 23.2	0.006
DBP (mmHg)	76.1 ± 55.4	75.3 ± 13.3	78.4 ± 102	n.s
Heart rate (bpm)	81.4 ± 25.1	80.3 ± 27.5	84.3 ± 17.1	0.031
Saturation (%)	96.6 ± 4.8	96.7 ± 5.2	96.5 ± 3.8	n.s
Respiratory rate	18.4 ± 7.9	18.1 ± 7.6	19.1 ± 8.7	n.s
GCS	14.9 ± 1.9	14.9 ± 2.2	14.9 ± 0.6	n.s
Body temperature (°C)	36.5 ± 1.0	36.4 ± 1.1	36.7 ± 0.9	< 0.001
*Signs and symptoms*				
Shock (%)	12 (1.3)	6 (0.9)	6 (2.2)	n.s
Palpitations (%)	27 (2.8)	26 (3.8)	1 (0.4)	0.002
Dyspnoea (%)	144 (15.1)	89 (13)	55 (20.6)	0.005
Asthenia (%)	66 (6.9)	47 (6.9)	19 (7.1)	n.s
Swollen legs (%)	86 (9)	62 (9)	24 (9)	n.s
Cough (%)	44 (4.6)	29 (4.2)	15 (5.6)	n.s
Chest pain (%)	73 (7.7)	57 (8.3)	16 (6)	n.s
Abdominal pain (%)	136 (14.3)	86 (12.5)	50 (18.7)	0.017
Vertigo (%)	39 (4.1)	29 (4.2)	10 (3.7)	n.s
Hemiplegia (%)	61 (6.4)	50 (7.3)	11 (4.1)	n.s
Headache (%)	27 (2.8)	21 (3.1)	6 (2.2)	n.s
Dysarthria (%)	62 (6.5)	45 (6.6)	17 (6.4)	n.s
Syncope (%)	69 (7.2)	48 (7)	21 (7.9)	n.s
Fall (%)	114 (12)	70 (10.2)	44 (16.5)	0.010
Altered mental status (%)	105 (11)	71 (10.3)	34 (12.7)	n.s
Vomit (%)	71 (7.5)	31 (4.5)	40 (15)	< 0.001
Diarrheal (%)	23 (2.4)	13 (1.9)	10 (3.7)	n.s
Convulsions (%)	9 (0.9)	8 (1.2)	1 (0.4)	n.s
*Comorbidities*				
Arterial hypertension (%)	565 (59.3)	402 (58.6)	163 (61)	n.s
Diabetes Mellitus (%)	211 (22.1)	144 (21)	67 (25.1)	n.s
COPD (%)	75 (7.9)	47 (6.9)	28 (10.5)	n.s
Atrial fibrillation (%)	166 (17.4)	112 (16.3)	54 (20.2)	n.s
Ischemic heart disease (%)	105 (11)	80 (11.7)	25 (9.4)	n.s
Ischemic stroke/TIA (%)	80 (8.4)	54 (7.9)	25 (9.7)	n.s
Heart failure (%)	29 (3)	26 (3.8)	3 (1.1)	n.s
Dementia/cognitive impairment (%)	69 (7.2)	44 (6.4)	25 (9.4)	n.s
Chronic liver disease / Cirrhosis (%)	22 (2.3)	15 (2.2)	7 (2.6)	n.s
Active cancer (%)	97 (10.2)	68 (9.9)	29 (10.9)	n.s
Epilepsy (%)	23 (2.4)	19 (2.8)	4 (1.5)	n.s
Dialysis (%)	11 (1.2)	8 (1.2)	3 (1.1)	n.s
*Chronic medications*				
ACEi /ARB (%)	386 (45.6)	283 (46.7)	103 (42.9)	n.s
ndCCB (%)	47 (5.6)	28 (4.6)	19 (7.9)	n.s
dCCB (%)	139 (16.4)	103 (17)	36 (15)	n.s
B-blockers (%)	330 (39)	244 (40.3)	86 (35.8)	n.s
Anticoagulants (%)	210 (24.8)	140 (23.1)	70 (29.2)	n.s
Antiplatelets (%)	301 (35.6)	215 (35.5)	86 (35.8)	n.s
Diuretics (%)	235 (27.8)	155 (25.6)	80 (33.3)	0.027
Corticosteroids (%)	73 (8.6)	46 (7.6)	27 (11.3)	n.s
Proton pump inhibitors (%)	340 (40.2)	235 (38.8)	105 (43.8)	n.s
Statins (%)	210 (24.8)	151 (24.9)	59 (24.6)	n.s
Benzodiazepines (%)	81 (9.6)	55 (9.1)	26 (10.8)	n.s
SSRI (%)	74 (8.7)	50 (8.3)	24 (10)	n.s
Anticonvulsants (%)	70 (8.3)	54 (8.9)	16 (6.7)	n.s
Antipsychotic (%)	48 (5.7)	36 (5.9)	12 (5)	n.s
Oral Hypoglycaemic Medications (%)	130 (15.4)	94 (15.5)	36 (15)	n.s
Insulin (%)	50 (5.9)	33 (5.4)	17 (7.1)	n.s
*Laboratory values*				
Blood urea nitrogen (mg/dl)	21 [16–29]	20 [15–27]	24.5 [18–34]	< 0.001
Direct bilirubin (mg/dl)	0.23 [0.16–0.36]	0.21 [0.14–0.32]	0.29 [0.20–0.39]	< 0.001
Total Bilirubin (mg/dl)	0.55 [0.36–0.86]	0.49 [0.32–0.78]	0.65 [0.46–1.01]	< 0.001
CK (UI/L)	75 [45–126]	75 [47–121]	76 [42–147]	n.s
CK-MB (ng/ml)	1.7 [1.2–2.9]	1.6 [1.2–3.0]	1.9 [1.22–2.9]	n.s
Creatinine (mg/dl)	1.3 ± 1.1	1.2 ± 1.1	1.4 ± 1.1	0.044
eGFR MDRD (ml/min)	68.5 ± 29.9	69.8 ± 28.1	65.3 ± 33.9	0.040
Haemoglobin (g/dl)	12.8 ± 4.1	12.9 ± 4.5	12.5 ± 3.0	n.s
White blood cells (× 10^3^/µL)	9.1 ± 5.3	7.9 ± 4.9	12.1 ± 5.1	< 0.001
Neutrophils %	72.3 ± 13.8	66.7 ± 12.1	86.6 ± 4.0	< 0.001
Lymphocytes %	17.9 ± 10.9	22.3 ± 9.7	6.7 ± 2.4	< 0.001
Monocytes%	5.9 ± 2.8	6.3 ± 2.9	4.8 ± 2.3	< 0.001
Eosinophils %	1.5 ± 2.1	1.8 ± 2.3	0.6 ± 0.7	< 0.001
Basophils %	0.4 ± 0.6	0.5 ± 0.7	0.2 ± 0.2	< 0.001
Platelet count (× 10^3^/µL)	228.8 ± 95.9	220.0 ± 82.6	251.6 ± 120.9	< 0.001
MPV (fl)	9.3 ± 1.1	9.3 ± 1.1	9.2 ± 1.1	n.s
Fibrinogen (mg/dl)	450.9 ± 112.2	433.6 ± 103.9	494.6 ± 120.2	< 0.001
D-Dimer (µg/L)	1147 [566–2566]	926 [468–1972]	1861 [1008–4190]	< 0.001
Blood glucose (ng/dl)	129.5 ± 59.4	121.2 ± 45.8	150.3 ± 81.0	< 0.001
Potassium (mEq/L)	4.3 ± 1.3	4.3 ± 1.5	4.2 ± 0.7	n.s
CRP (mg/dl)	0.78 [0.17–4.46]	0.43 [0.13–2.17]	4.24 [0.67–11.8]	< 0.001
Sodium (mEq/L)	138.6 ± 13.7	138.9 ± 11.5	137.8 ± 18.0	n.s
LDH (UI/L)	251.8 ± 141.1	235.2 ± 115.7	293.2 ± 183.9	< 0.001
GOT / AST (UI/L)	20 [16–27]	19 [16–25]	22 [15–35]	< 0.001
GPT / ALT (UI/L)	13 [10–21]	13 [9–19.5]	15 [10–25]	< 0.001

*ACEi* angiotensin-converting enzyme inhibitors, *ARB* angiotensin receptor blockers, *CK* creatine kinase, *CK-MB* creatine kinase-MB, *COPD* chronic obstructive pulmonary disease, *CRP* C-reactive protein, *DBP* diastolic blood pressure, *dCCB* dihydropyridine calcium channel blockers, *eGFR* estimated glomerular filtration rate, *GCS* Glasgow coma scale, *GOT/AST* glutamic-oxalacetic transaminase**/**aspartate transaminase, *GPT/ALT* glutamic-pyruvic transaminase/alanine transaminase, *LDH* lactate dehydrogenase, *MPV* mean platelet volume, *ndCCB* non-dihydropyridine calcium channel blockers, *SBP* systolic blood pressure, *SSRI* selective serotonin reuptake inhibitors, *TIA* transitory ischemic attack

When admitted to the ED, patients with NLR > 8, compared to those with a NLR < 8, were older and characterized by a more serious triage code (only 1% had a triage code = 4, while 72.6% had a triage code ≤ 2) and by an increased heart rate and body temperature. They more often presented with dyspnea, abdominal pain, falls and vomiting. Regarding comorbidities, there were no significant differences between the two groups.

Patients with a NLR > 8 were more frequently on chronic therapy with diuretics; they are also characterized by an increase in urea, creatinine, white blood cells, neutrophils, fibrinogen, D-dimer, glycemia, CRP, lactate dehydrogenase (LDH) and by a decrease in the estimated glomerular filtration rate (eGFR), of lymphocytes and monocytes (Table 1).

### Mortality

A total of 27 patients were lost during follow-up, therefore, the survival analysis was performed on a cohort of 926 patients. In total, 142 (15.3%) patients died in-hospital or during the 30-day follow-up. Those patients who died during the follow-up were characterized by a more severe triage code, they were older and presented with a higher respiratory rate. In terms of symptoms and signs, the occurrence of an adverse outcome was more often associated with shock, dyspnea, swollen legs, vertigo, altered mental status, vomiting and diarrhea at presentation (Table 2). In our cohort, COPD, atrial fibrillation, dementia and active cancer were the comorbidities more commonly found in those patients who died during follow-up. The remaining characteristics stratified by vital status are presented in Table 2.

**Table 2:** Population characteristics according to vital status

Variable	Alive patients (*n* = 784)	Dead patients (*n* = 142)	*P* value
Triage code 1	146(18.5%)	51(36.2%)	< 0.001
2	342(43.6%)	62(44%)	
3	256(32.7%)	29(19.9%)	
4	40(5.1%)	0%	
Women	385 (49.1)	71 (50)	n.s
Age	78.3 ± 7.8	83.5 ± 7.8	< 0.001
SBP (mmHg)	135.0 ± 22.7	125.4 ± 23.3	< 0.001
DBP (mmHg)	77.3 ± 60.7	69.6 ± 12.2	n.s
Heart rate	81.2 ± 26.2	83.7 ± 18.6	n.s
Oxygen saturation (%)	96.7 ± 5.2	96.2 ± 3.1	n.s
Respiratory rate	18.0 ± 6.1	20.5 ± 14.4	0.002
GCS	14.9 ± 0.7	14.9 ± 4.8	n.s
Body temperature (°C)	36.4 ± 1.1	36.6 ± 0.8	n.s
*Signs and symptoms*			
Shock (%)	6 (0.8)	6 (4.2)	0.005
Palpitations (%)	26 (3.3)	1 (0.7)	n.s
Dyspnoea (%)	104 (13.3)	36 (25.4)	0.001
Asthenia (%)	52 (6.6)	10 (7)	n.s
Swollen legs (%)	63 (8)	21 (14.8)	0.016
Cough (%)	36 (4.6)	7 (4.9)	n.s
Chest pain (%)	61 (7.8)	6 (4.2)	n.s
Abdominal pain (%)	115 (14.7)	17 (12)	n.s
Vertigo (%)	37 (4.7)	1 (0.7)	0.021
Hemiplegia (%)	46 (5.9)	14 (9.9)	n.s
Headache (%)	22 (2.8)	4 (2.8)	n.s
Dysarthria (%)	49 (6.3)	11 (7.7)	n.s
Syncope (%)	63 (8)	6 (4.2)	n.s
Fall (%)	91 (11.6)	19 (13.4)	n.s
Altered mental status (%)	68 (8.7)	35 (24.6)	< 0.001
Vomit (%)	54 (6.9)	16 (11.3)	n.s
Diarrheal (%)	15 (1.9)	8 (5.7)	0.016
Convulsions (%)	7 (0.9)	2 (1.4)	n.s
*Comorbidities*			
Arterial hypertension (%)	464 (59.2)	85 (59.9)	n.s
Diabetes Mellitus (%)	171 (21.8)	32 (22.5)	n.s
COPD (%)	55 (7)	17 (12)	n.s
Atrial fibrillation (%)	126 (16.1)	34 (23.9)	n.s
Ischemic heart disease (%)	90 (11.5)	13 (9.2)	n.s
Ischemic stroke/TIA (%)	66 (8.4)	13 (9.2)	n.s
Heart failure (%)	26 (3.3)	3 (2.1)	n.s
Dementia / Cognitive impairment (%)	48 (6.1)	19 (13.4)	0.004
Chronic liver disease / Cirrhosis (%)	16 (2)	5 (3.5)	n.s
Active cancer (%)	73 (9.3)	22 (15.5)	0.034
Epilepsy (%)	19 (2.4)	4 (2.8)	n.s
Dialysis (%)	8 (1)	3 (2.1)	n.s
*Chronic medications*			
ACEi /ARB (%)	334 (47.9)	45 (35.2)	0.009
ndCCB (%)	42 (6)	4 (3.1)	n.s
dCCB (%)	117 (16.8)	16 (12.5)	n.s
B-blockers (%)	258 (37)	62 (48.4)	0.018
Anticoagulants (%)	164 (23.5)	40 (31.3)	n.s
Antiplatelets (%)	248 (35.6)	48 (37.5)	n.s
Diuretics (%)	181 (26)	46 (35.9)	0.024
Corticosteroids (%)	54 (7.7)	17 (13.3)	n.s
Proton pump inhibitors (%)	272 (39)	63 (49.2)	0.039
Statins (%)	178 (25.5)	29 (22.7)	n.s
Benzodiazepines (%)	62 (8.9)	16 (12.5)	n.s
SSRI (%)	62 (8.9)	9 (7)	n.s
Anticonvulsants (%)	54 (7.7)	14 (10.9)	n.s
Antipsychotic (%)	32 (4.6)	14 (10.9)	0.010
Oral Hypoglycaemic Medications (%)	105(15.1)	19 (14.8)	n.s
Insulin (%)	34 (4.9)	14 (10.9)	0.012
*Laboratory values*			
NLR > 8	186 (23.7)	75 (52.8)	< 0.001
Blood urea nitrogen (mg/dl)	20 [15–28]	26 [19–43]	< 0.001
Direct Bilirubin (mg/dl)	0.23 [0.16–0.34]	0.32 [0.20–0.61]	< 0.001
Total Bilirubin (mg/dl)	0.53 [0.35–0.81]	0.76 [0.44–1.39]	< 0.001
CK (UI/L)	75 [47–125]	68 [35–123]	n.s
CK-MB (ng/ml)	1.7 [1.3–2.8]	2.6 [1.2–4.3]	n.s
Creatinine (mg/dl)	1.2 ± 1.0	1.5 ± 1.4	0.006
eGFR MDRD (ml/min)	68.9 ± 28.4	64.8 ± 37.3	n.s
Haemoglobin (g/dl)	12.9 ± 4.4	11.8 ± 2.2	0.002
White blood cells (× 10^3^/µL)	8.9 ± 5.2	10.2 ± 5.8	0.009
Neutrophils %	71.1 ± 13.8	79.4 ± 11.5	< 0.001
Lymphocytes %	18.9 ± 10.9	12.5 ± 9.4	< 0.001
Monocytes%	6.0 ± 2.9	5.3 ± 2.4	0.009
Eosinophils %	1.6 ± 2.2	0.9 ± 1.3	0.001
Basophils %	0.4 ± 0.6	0.3 ± 0.2	0.005
Platelet count (× 10^3^/µL)	227.2 ± 89.4	239.7 ± 129.9	n.s
MPV (fl)	9.3 ± 1.0	9.6 ± 1.1	< 0.001
Fibrinogen (mg/dl)	449.6 ± 110.1	460.5 ± 127.5	n.s
D-Dimer (µg/L)	1066 [526–2256]	1863 [907–4400]	< 0.001
Blood glucose (ng/dl)	127.2 ± 55.4	143.0 ± 76.8	0.004
Potassium (mEq/L)	4.3 ± 1.4	4.3 ± 0.8	n.s
CRP (mg/dl)	0.59 [0.15–3.8]	2.9 [0.7–10.1]	< 0.001
Sodium (mEq/L)	139.1 ± 14.6	136.1 ± 7.9	0.020
LDH (UI/L)	237.5 ± 93.2	326.5 ± 279.4	< 0.001
GOT / AST (UI/L)	20 [15–26]	22 [16–34]	0.021
GPT / ALT (UI/L)	13 [10–20]	13 [8–23]	n.s

*ACEi* angiotensin-converting enzyme inhibitors, *ARB* Angiotensin receptor blockers, *CK* creatine kinase, *CK-MB* creatine kinase-MB, *COPD* chronic obstructive pulmonary disease, *CRP* C-reactive protein, *DBP* diastolic blood pressure, *dCCB* dihydropyridine calcium channel blockers, *eGFR* estimated glomerular filtration rate, *GCS* Glasgow coma scale, *GOT/AST* glutamic-oxalacetic transaminase**/**aspartate transaminase, *GPT/ALT* glutamic-pyruvic transaminase/alanine transaminase, *LDH* lactate dehydrogenase, *MPV* mean platelet volume, *ndCCB* non-dihydropyridine calcium channel blockers, *SBP* systolic blood pressure, *SSRI* selective serotonin reuptake inhibitors, *TIA* transitory ischemic attack

### Association of NLR with mortality

**Fig. 1:**
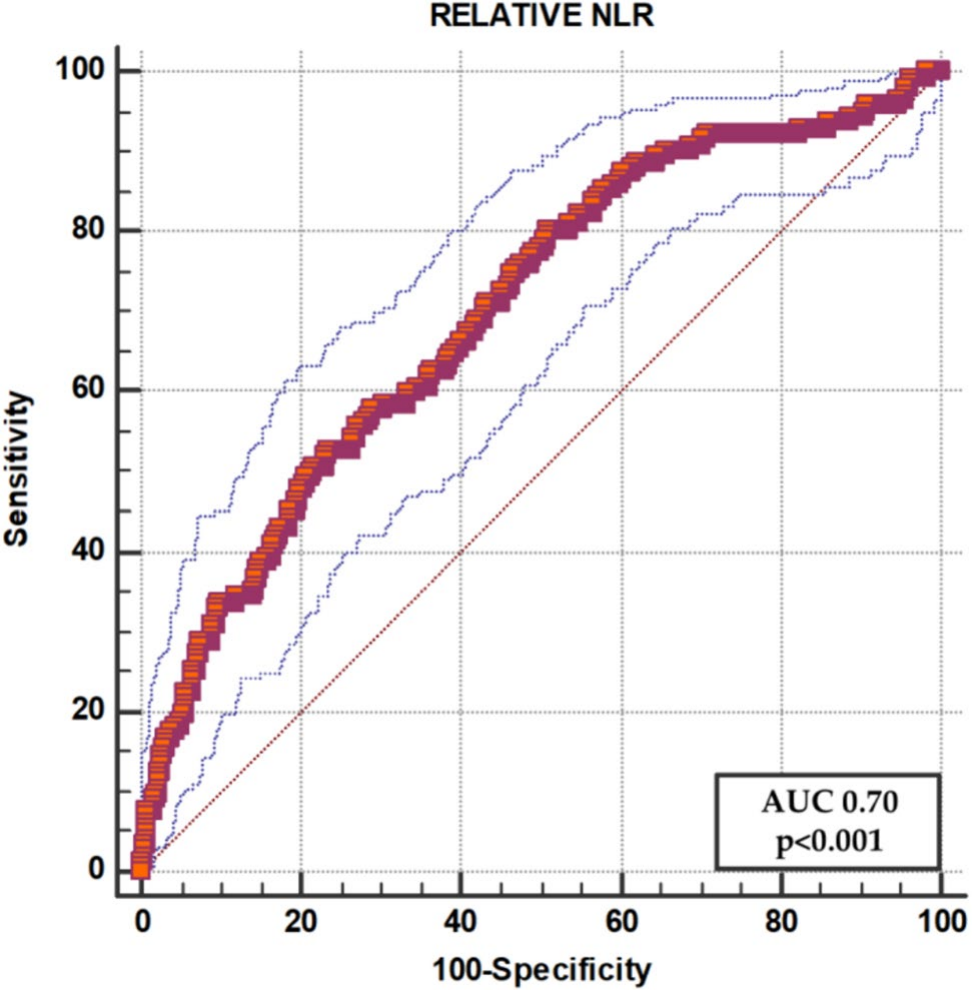
ROC analysis for all-cause mortality. *NLR* Neutrophil to lymphocyte ratio

In our cohort, the NLR as a continuous value reached an AUC of 0.70 (95%CI 0.67–0.73 *p* < 0.001) for the outcome of all-cause mortality at 30 days (on 926 patients for whom survival data were available) (Fig. 1).

This value was consistent in the group of women (*n* = 456, 71 deaths) with an AUC of 0.71, 95%CI 0.67–0.75 and a best cut-off of > 4.47. The optimal calculated cut-off for this variable in our cohort was 8.

To analyze the relationship between all-cause mortality and different predictors we built a logistic regression model. The OR of having a NLR > 8 was 2.563 (95%CI 1.595–4.118) with a p < 0.001. Another strong association with the outcome was found to be an altered mental status at presentation with an OR of 3.610 (95%CI 1.976–6.596). The results for the other predictors are shown in Table 3.

**Table 3:** Multivariable logistic regression analysis of factors associated with all-cause mortality

	Odds ratio	95% Confidence interval		*p* value
		Lower	Upper	
Relative NLR > 8	2.563	1.595	4.118	< 0.001
Age	1.055	1.024	1.087	< 0.001
Systolic blood pressure	0.989	0.979	0.999	0.037
Respiratory rate	1.023	1.002	1.044	0.029
Altered mental status	3.610	1.976	6.596	< 0.001
Active cancer	1.987	1.024	3.853	0.042
LDH	1.002	1.001	1.004	0.005
MPV	1.273	1.021	1.589	0.032

*LDH* lactate dehydrogenase, *MPV* mean platelet volume, *NLR* neutrophil to lymphocyte ratio

## Discussion

In this analysis including almost 1000 patients, we have described the characteristics of elderly patients presenting to the ED for acute medical conditions according to their NLR at presentation. We found that NLR is an independent predictor of mortality during 30 days of follow-up. In particular, we found an optimized cut-off of NLR > 8 specific for acute medical patients aged > 65 years, which differs from previous studies. Patients admitted with a NLR > 8 had specific characteristics with a generally more severe disease presentation.

In our cohort, the mortality rate at 30 days was 15.3%. In the literature, we found very heterogeneous reports about short-term mortality in elderly patients admitted to the ED, ranging from 8 to 25% [2, 23, 24].

Byrne DG et al. found in a cohort of 23,114 patients that 25.8% were over the age of 75 years. For this group, the 30-day mortality was 20.7% compared with 4.5% for younger patients [25]. In these studies, the main symptoms associated with adverse outcomes were delirium, hypoxemia, unconsciousness at presentation, female sex, age [23, 24]. Our results are in line with those previous findings, showing that respiratory rate, altered mental status, and systolic blood pressure were associated with an increased risk of mortality.

Our main finding relies on the clinically useful predictive value of the NLR in acute elderly medical patients. We used an optimized cut-off for NLR > 8, providing an AUC of 0.70. This value is in line with many other clinical scores currently used in clinical practice [26, 27].

In our cohort, 28% of patients had a NLR > 8. These patients had more severe symptoms and signs at presentation. Patients with a NLR > 8 showed a 2.5-fold higher risk of dying during the follow-up.

Different cut-off values for the NLR have been proposed in recent publications, depending on the clinical setting and underlying pathology [8]. Regolo M. et al. showed that in-hospital mortality in COVID-19 patients is predicted by a NLR > 11.38 (AUC = 0.77, *p* < 0.001) [26].

In patients older than 80 years hospitalized a NLR on admission > 2.97 (sensitivity of 92.6% and specificity of 52.5%, AUC = 0.714, *p* = 0.001) was an independent predictor of all-cause 3-month mortality [27]. Similarly, in a cohort of more than 5000 hospitalized geriatric patients an NLR higher than 7.95 was associated with in-hospital mortality with an AUC of 0.707 (0.686–0.728) [28]. This is in line with our results, showing that the NLR could be useful in risk stratification for all-cause mortality in elderly patients presenting to the ED for medical conditions. Furthermore, a test that could quickly classify nearly 30% of such patients as low-risk of short term mortality could be an additional support to emergency physicians for a safe discharge.

This laboratory value can be obtained quickly as part of the laboratory routine and is inexpensive. Given the lack of systematic risk stratification strategies, the proposal of the simple NLR in the setting of the emergency may be particularly useful.

Another interesting finding is the association of MPV with mortality. This is in line with previous studies in literature. In fact, an increased MPV seems to correlate with higher mortality in patients suffering from myocardial infarction, critically ill patients and in septic patients [29–31]. This could reflect a systemic inflammatory response, as inflammation, endothelial dysfunction and thrombotic conditions may alter platelet size [32].

Some limitations should be considered when interpreting these findings. First, patients with traumatic injuries were excluded from this study, therefore, we cannot generalize our findings to those patients. Then, the value of NLR can be falsely increased in some patients. As described by Karakonstantis et al., factors such as age, exogenous steroid intake, endogenous sexual hormones, active hematological disorders, such as leukemia, cytotoxic or granulocyte colony-stimulating factor chemotherapies, and HIV can determine an increase of the NLR [33].

In conclusion, NLR is a simple tool that is associated with the severity of presentation and all-cause mortality; therefore, elderly patients with a NLR > 8 should be early identified as being at higher risk of adverse outcomes and promptly managed.

## Data Availability

The data that support the findings of this study are available from the authors but restrictions apply to the availability of these data and so are not publicly available. Data are, however, available from the authors upon reasonable request and with permission from Ethical Committee.
